# Glycobiology Aspects of the Periodontal Pathogen *Tannerella forsythia*

**DOI:** 10.3390/biom2040467

**Published:** 2012-10-11

**Authors:** Gerald Posch, Gerhard Sekot, Valentin Friedrich, Zoë A. Megson, Andrea Koerdt, Paul Messner, Christina Schäffer

**Affiliations:** Department of NanoBiotechnology, NanoGlycobiology Unit, Universität für Bodenkultur Wien, Muthgasse 11, A-1190 Vienna, Austria; Email: gerald.posch@boku.ac.at (G.P.); valentin.friedrich@boku.ac.at (V.F.); zoe.megson@boku.ac.at (Z.A.M.); andrea.koerdt@boku.ac.at (A.K.)

**Keywords:** Biofilm, general *O*-glycosylation system, Gram-negative oral pathogen, glycosidases, S-layer glycoproteins, *Tannerella forsythia*, virulence

## Abstract

Glycobiology is important for the periodontal pathogen *Tannerella forsythia*, affecting the bacterium’s cellular integrity, its life-style, and virulence potential*.* The bacterium possesses a unique Gram-negative cell envelope with a glycosylated surface (S-) layer as outermost decoration that is proposed to be anchored via a rough lipopolysaccharide. The S-layer glycan has the structure 4‑Me*O*-b-Man*p*NAcCONH_2_-(1→3)-[Pse5Am7Gc-(2→4)-]-b-Man*p*NAcA-(1→4)-[4-Me*O*-a-Gal*p*-(1→2)-]-a-Fuc*p*-(1→4)-[-a-Xyl*p*-(1→3)-]-b-Glc*p*A-(1→3)-[-b-Dig*p*-(1→2)-]-a-Gal*p* and is linked to distinct serine and threonine residues within the D(S/T)(A/I/L/M/T/V) amino acid motif. Also several other *Tannerella* proteins are modified with the S‑layer oligosaccharide, indicating the presence of a general *O*‑glycosylation system. Protein *O*‑glycosylation impacts the life-style of *T. forsythia* since truncated S-layer glycans present in a defined mutant favor biofilm formation. While the S‑layer has also been shown to be a virulence factor and to delay the bacterium's recognition by the innate immune system of the host, the contribution of glycosylation to modulating host immunity is currently unraveling. Recently, it was shown that *Tannerella* surface glycosylation has a role in restraining the Th17-mediated neutrophil infiltration in the gingival tissues. Related to its asaccharolytic physiology, *T. forsythia* expresses a robust enzymatic repertoire, including several glycosidases, such as sialidases, which are linked to specific growth requirements and are involved in triggering host tissue destruction. This review compiles the current knowledge on the glycobiology of *T. forsythia*.

## 1. Introduction to *Tannerella forsythia*

### 1.1. Occurrence of *T. forsythia*

It has been estimated that nearly 700 bacterial taxa, phylotypes and species can colonize the oral cavity of humans [1]. Many of them trigger periodontal diseases which are multifactorial infections implicating interactions with host tissues and cells. These may lead to destruction of the periodontal structures, including the tooth-supporting tissues, alveolar bone, and periodontal ligament [2]. Frequently, the trigger for the initiation of periodontal diseases is the presence of complex microbial biofilms that colonize the sulcular regions between the tooth surface and the gingival margin [3,4]. Recently, the link between oral microbial communities with the change from health to disease was investigated, leading to a classification of the microbiota into bacterial consortia (‘complexes‘) that occur together and are associated with the sequence of colonization on the tooth surface as well as with disease severity [5,6,7]. The ‘red complex’, which has been classified as a late colonizer in multispecies biofilm development, comprises species that are considered periodontal pathogens [4]; these are *Porphyromonas gingivalis*, *Treponema denticola*, and *Tannerella forsythia*.

### 1.2. Description of *T. forsythia*

#### 1.2.1. Taxonomic Affiliation

*T. forsythia* was first isolated in the mid-1970s from subjects with progressing advanced periodontitis and described as ‘fusiform *Bacteroides*’ by Tanner *et al.* [8]. Initially, its taxonomic affiliation was unclear because it did not resemble described species of oral or enteric Gram-negative anaerobic rods [9]. The phylogeny of oral *Bacteroides* species in the *Cytophaga–Flavobacterium–Bacteroides* family was reorganized after *Bacteroides forsythus* had been described and eventually clarified in the phylogenetic studies comparing 16S rRNA sequence data [10]. Subsequently, *B. forsythus* was affiliated to the genus *Tannerella* [11]. Here it was first formally classified to *Tannerella forsythensis* but then reclassified to *T. forsythia* [12].

Recent taxonomic analyses have revealed that *Bacteroides*, *Porphyromonas* and *Tannerella* are all contained in the order *Bacteroidales* and that *Porphyromonas* and *Tannerella* are phylogenetically even more closely related, as both are affiliated to the *Porphyromonadaceae* family [13].

A preliminary rapid identification of human-derived *T. forsythia* strains can be based on the following eight criteria [14]: positive activity for (i) a‑glucosidase; (ii) b-glucosidase; (iii) sialidase; (iv) trypsin-like enzyme; (v) negative indole production; (vi) requirement for *N*‑acetylmuramic acid; (vii) colonial morphology; and (viii) Gram-stain morphology from blood agar medium deficient in *N*-acetylmuramic acid.

The full genome sequence of *T. forsythia* ATCC 43037 is available through the Oral Pathogen Sequence Databases at Los Alamos National Laboratory Bioscience Division [15]. The genome consists of 3,405,543 base pairs with 3,034 predicted open reading frames.

#### 1.2.2. *T. forsythia* as a Periodontal Pathogen

*T. forsythia* meets the criteria for periodontal pathogens postulated by Socransky [16] and Socransky *et al*. [17], (i) because this bacterium is present in increased levels in periodontitis [17]; (ii) there is evidence for host response to its antigens [18,19,20]; (iii) it is able to cause disease in animal models [21,22,23]; and (iv) it expresses virulence factors that can potentially contribute to the disease process [24]. Among the characterized *T. forsythia* virulence factors related to the field of glycobiology are a sialidase [25], an a-D-glucosidase and an *N*-acetyl-b-glucosaminidase [26], as well as the glycosylated surface (S-) layer [27,28,29].

Despite the growing evidence implicating this bacterium in the pathogenesis of periodontitis, it is up to now a rather poorly studied organism, a fact that can be attributed to its fastidious growth requirements as well as to the lack of molecular tools for genetic manipulation [24].

Recent evidence suggests that glycobiology is important for defining the life of the periodontal pathogen *T. forsythia*. This review compiles the current state of knowledge about the glycobiology aspects of *T. forsythia*, with relevance for the bacterium’s cellular integrity, its life-style and metabolism as well as its virulence potential.

## 2. Cellular Integrity

### 2.1. S-Layer Glycosylation of *T. forsythia*

#### 2.1.1. S-Layers in General

Many bacteria from all phylogenetic lineages are covered by regularly arrayed superficial layers, termed S-layers. The protomeric units of S-layers usually consist of high-molecular-mass proteins or glycoproteins [30,31]. Particularly in Gram-negative bacteria it has been difficult to isolate these structures in a form which truly represents what might occur in the living organism, although some attempts have been made [32,33,34]. Different functions, such as protective covering, molecular sieve and ion trap, phage receptor, providing an adhesion and surface recognition mechanism, as well as involvement in mediation of virulence, have been attributed to S-layers [30,31].

#### 2.1.2. S-Layer Ultrastructure of *T. forsythia*

About 25 years ago, Kerosuo [35] reported for the first time on the ultrastructure of the *T. forsythia* ATCC 43037^T^ S-layer. Later, Sabet *et al.* [27] published their findings regarding the isolation, purification, and initial studies on the virulence potential of the S-layer from *T. forsythia* strains. SDS-PAGE analysis revealed the presence of two high molecular-mass, glyco-positive protein bands [36,37,38], which were later confirmed by our research group to be the components of the S-layer [39]. The ~135-kDa *Tannerella* S-layer protein TfsA and the ~152-kDa S-layer protein TfsB are encoded by the genes *tfsA* (TF2661-2662) and *tfsB* (TF2663), respectively, which are co-transcribed from a single promoter [38]. The two S-layer proteins share 24% amino acid similarity. They do not show overall homology to any other S-layer protein sequence deposited in databases, except for their *C*‑terminal regions, which have profound similarity to putative S‑layer glycoproteins of the phylogenetically closely related bacterium *Bacteroides distasonis* [40]. The *Tannerella* S‑layer proteins exhibit C‑terminal sequence similarity to the CTD (C-Terminal Domain) family proteins of *Porphyromonas gingivalis* which supports the assumption of a novel CTD *Bacteroidales* secretion pathway [41,42].

Our interest in the S-layer of *T. forsythia* was aroused by the facts that (i) it represents the first glycosylated S-layer of a Gram-negative organism and (ii) it is structurally unique due to the simultaneous presence of two S-layer proteins [38]. Interestingly, in periodontal lesions, another S‑layer-carrying bacterium, namely *Campylobacter rectus* is found. This organism is thought to be capable of inducing pro-inflammatory cytokines and its S-layer may temper this response to facilitate the survival of *C. rectus* at the site of infection [43].

**Figure 1:**
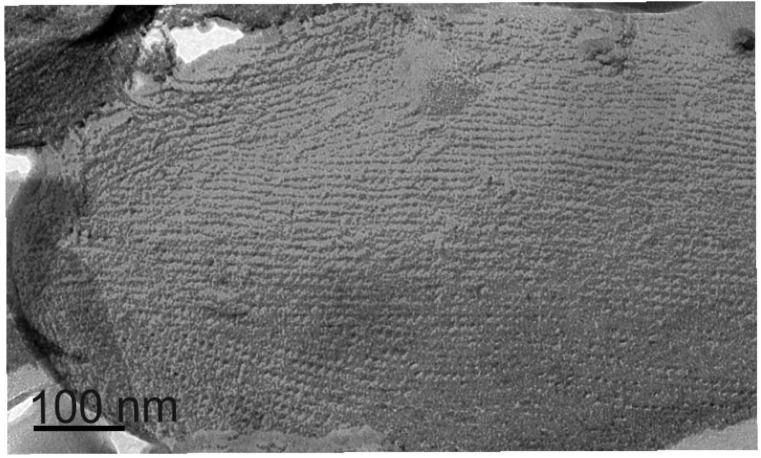
Freeze-etched and platinum-carbon shadowed preparation of a *T. forsythia* cell showing the square S-Layer lattice with a lattice spacing of approximately 10 nm × 10 nm.

Recently, Sekot *et al.* [39] extended the previous structural characterization of the *T. forsythia* S‑layer [35] by a combined ultrastructural/immunological approach, showing that the two S-layer glycoproteins TfsA-GP and TfsB-GP are intercalated to form a monolayer on the cell surface of *T. forsythia*. The lattice spacing for the square S-layer lattice was determined to be 10.1 ± 0.7 nm (Figure 1) in freeze-etching, freeze-drying, and negative staining experiments, and a thickness of the layer of approximately 22 nm was found in ultrathin sections.

In that study, sheared flagella with intact hook regions have been identified by freeze-etching [39], which somehow challenges the description of *T. forsythia* as a non-motile species [44]. However, their role in bacterial motility and a possible impact on co-aggregation of *T. forsythia* with other species in the biofilm is still unclear. In this context it should also be mentioned that according to a recent partial annotation of the open reading frames of the *T. forsythia* genome, homologous genes for hook and other pilus/flagella forming units have not been identified [45].

#### 2.1.3. Glycosylation of the *T. forsythia* S-layer Proteins TfsA and TfsB

As mentioned above, Lee *et al.* [38] inferred from SDS-PAGE that the two *T. forsythia* S-layer proteins are glycosylated. However, no further compositional or structural details were provided. In our laboratory we focused the efforts on the characterization of the glycoproteome of *T. forsythia*.

By SDS-PAGE, glycosylation of the two S-layer proteins of *T. forsythia*, the 153-kDa TfsA-GP and the 180-kDa TfsB-GP (calculated molecular masses), could be confirmed. In-gel reductive b-elimination of purified S-layer glycoproteins was employed to release the glycans from the protein backbone [29]. Mass spectrometric analysis revealed a dominant glycan structure of 1,621 Da. Upon CID fragmentation analysis a hetero-oligomer consisting of eight different sugar residues was observed. Mass increments for one pentose, one deoxyhexose, three uronic acids (modified or free), one methylhexose, and one reduced hexose in addition to one, yet non-described, 361-Da sugar residue were identified. Further, some of the oligosaccharides were substituted by one dideoxyhexose and one additional deoxyhexose. Interestingly, the glycans released from either S-layer glycoprotein showed identical glycan mass profiles, indicating the presence of a uniform glycosylation pattern.

**Figure 2:**
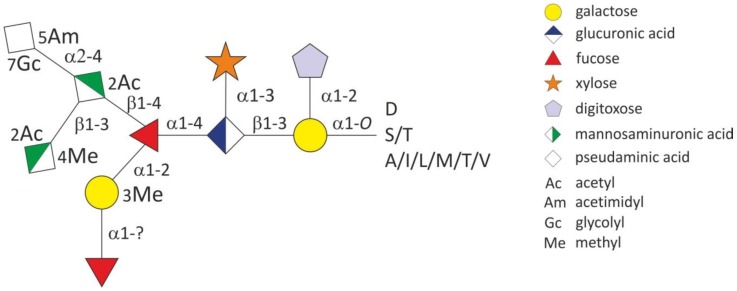
Schematic drawing of the structure of the abundant *O*-glycan in *T. forsythia*. Modified from [29].

NMR spectroscopy of the glycan samples was used to identify the methyl-hexose as methyl-galactose and the two *N*-acteylhexosaminuronic acid residues as *N*-acetylmannosaminuronic acid and as *O*-methyl-*N*-acetylmannosaminuronic acid, respectively. The dideoxyhexose that was already observed in the MS/MS fragmentation profile was identified as a digitoxose residue. The identity of the 361-Da sugar residue, however, remained unclear even after high resolution MS/MS and NMR analysis of the intact *O*-glycan. In a screen of nucleotide-activated sugars from the bacterial cytoplasm large amounts of CMP-activated substance matching the 361-Da unit were observed. This was indicative for the presence of an a-keto sugar. Combined NMR analyses of the purified DMB (1,2-diamino-4,5-methylene dioxybenzene dihydrochloride)-labelled 361-Da sugar residue and of the complete oligosaccharide revealed the presence of a non‑2-ulosonic acid carrying two substituents on the amino functions at carbons 5 and 7. Comparison of all recorded NMR spectroscopic data of the *O*-glycan with those of the isolated C_14_H_25_O_9_N_3_ compound showed good concordance. The configuration of the non-2-ulosonic acid has been proven by the few detectable coupling constants and NOEs and is in better accordance with those of a pseudaminic acid residue than with those of legionaminic acid. The whole C_14_H_25_O_9_N_3_ compound can hence be considered as Pse5Am7Gc, where Am is an amidinyl and Gc a glycolyl group [29]. The structure of the highly complex decaglycan of *T. forsythia* including the so far determined glycosidic linkages is given in Figure 2. The heterosaccharide is *O*‑glycosidically linked via the reducing-end a‑galactose to serine and threonine residues of both the TfsA and TfsB S‑layer proteins. Interestingly, all identified glycosylation sites match the D(S/T)(A/I/L/M/T/V) three-amino acid motif that was recently described for the general protein *O*-glycosylation system in *B. fragilis* [46].

### 2.2. General O-Glycosylation System of *T. forsythia*

#### 2.2.1. *O*-Glycoproteins of *T. forsythia*

Carbohydrate-stained SDS-PAGE gels of *T. forsythia* whole cell lysates indicated, besides the S-layer glycoproteins, the presence of several other glycosylated proteins in the molecular mass range between 60 and 250 kDa [29]. Some of these bands were also reactive with the fucose-specific *Aleuria aurantia* lectin, which readily detects the S-layer glycan. Upon closer examination, all bands were shown to carry the same glycosylation profile as the S-layer glycoprotein bands, with slight variations detected in their relative ratios and in *O*-methylation of the mannosaminuronamide residue. This demonstrates that the S-layer *O*-glycosylation system is also involved in the glycosylation of other *T. forsythia* proteins [29].

Among these proteins are the predicted outer-membrane proteins TF2339 and its paralog TF1259, as well as the predicted lipoproteins TF1056 and TF0091. The former two proteins exhibit similarity to the CTD family of *P. gingivalis* [41,42]; the latter show similarity to TonB-dependent receptor associated proteins [47]. Interestingly, all of these glycoproteins are antigenic upon probing with an antiserum raised against a *T. forsythia* outer membrane preparation [45]. The finding that several abundant proteins in *T. forsythia* are modified with the S‑layer glycan is fueled by the recent identification of a rich outer membrane glycoproteome in *T. forsythia* [45].

#### 2.2.2. Theoretical analysis of protein *O*‑glycosylation in *T. forsythia*

Initial information about glycosylation in *T. forsythia* was provided by the description of a so-called exopolysaccharide operon [48], of which WecC (TF2055) coding for a predicted UDP-*N*-acetylmannosaminuronic acid dehydrogenase is part of. Closer inspection of that genomic region revealed the presence of a 6.8‑kb gene locus of *T. forsythia* spanning TF2055-TF2049, encoding in addition to WecC (TF2055), a predicted UDP-*N*-acetylglucosamine 2-epimerase (NeuC, TF2054), three predicted glycosyltransferases (TF2053; TF2050; TF2049), a predicted acetyltransferase (TF2052) and one ORF with yet unassigned function (TF2051) [29].

Since deletion of TF2055 caused truncation of S-layer protein glycans [48] by lacking the 809-Da Pse-containing trisaccharide side branch [29], it is evident that this genomic region carries crucial information for proper *O*‑glycan assembly (see Section 3.2. and Section 6.2.). Interestingly, similar glycosylation loci are also present in other phylogenetically related species, including, for instance, *Bacteroides fragilis* NCTC 9343, *Bacteroides thetaiotaomicron* VPI 5492, *Bacteroides uniformis* ATCC 8492, *Porphyromonas gingivalis* ATCC 33277, and *Parabacteroides distasonis* ATCC 8503.

### 2.3. Lipopolysaccharide (LPS) of *T. forsythia*

LPS is an intrinsic feature of Gram-negative bacteria where it is located in the outer leaflet of the outer membrane [49]. So far, structure-function studies on the LPS from *T. forsythia* do not exist but are considered essential to further understand the bacterium’s pathogenesis.

Currently, we are analyzing the LPS from *T. forsythia* and first results indicate that in the wild-type organism, an R-form LPS is present (G. Posch, O. Andrukhov, B. Lindner, P. Messner, E. Vinogradov, O. Holst, C. Schäffer, manuscript in preparation). Currently it is unknown, if LPS biosynthesis and the S-layer glycosylation pathway have cross-points. In a recent comparison of the LPS isolated from *T. forsythia* wild-type and Δ*wecC* cells by SDS-PAGE, no detectable effect of WecC on the chemical nature of LPS was observed [50].

**Figure 3:**
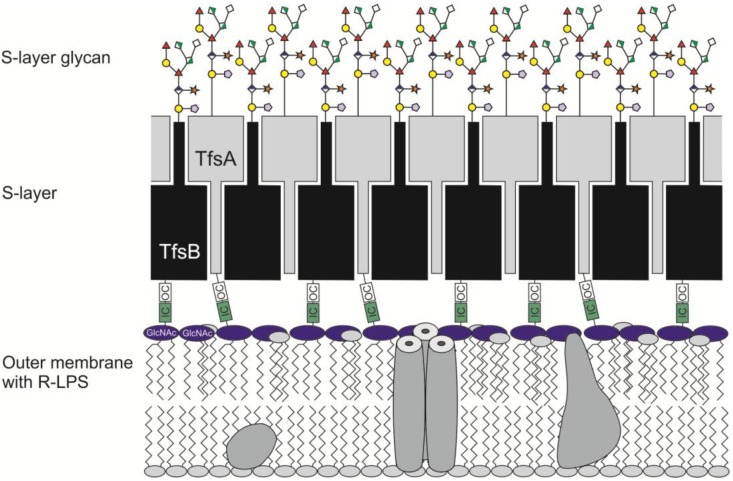
Theoretical model of the cell envelope architecture of *T. forsythia*. IC (inner core) and OC (outer core) represent partial structures of the rough LPS. Not to scale.

In this context it is interesting to note that in *Aeromonas salmonicida*, an R-form LPS is assumed to serve as an anchor with defined length for attaching the S-layer protein to the outer membrane [51] (Figure 3). Considering that both *T. forsythia* S‑layer proteins TfsA and TfsB have been classified as CTD-proteins [45], it should be noted that in a recent study with *P. gingivalis* it has been shown that LPS attaches to this class of proteins. It was suggested that LPS deacetylation is part of the co-ordinated secretion of LPS and CTD-proteins by a novel secretion and attachment system [42].

## 3. Virulence Potential of the Glycosylated S‑Layer of *T. forsythia*

### 3.1. Virulence and Glycosylation in General

Virulence is the relative ability of an organism to cause disease or to interfere with a metabolic or physiological function of its host. Thus, virulence factors can have a multitude of functions, including the ability to induce microbe-host interactions such as attachment, to invade the host, to grow in the confines of a host cell, and to evade or even interfere with host defense mechanisms [4].

Cell surface glycosylation and, in that context, specifically glycosylation of proteins, might serve specific functions in infection and interaction with host tissues [52] as well as modulation of immune responses during pathogenesis [53,54]. Via interaction with components of the immune system, the glycan rather than the amino acid residues represent immuno-dominant epitopes that are recognized by blocking antibodies or modulate the immune response [52].

### 3.2. Immunological Data of *T. forsythia*

The S-layer glycoproteins TfsA-GP and TfsB-GP of *T. forsythia* are strongly antigenic, mediate hemagglutination as well as adherence to- and invasion of KB cells [38,55]. While the levels of IgG antibody against the S‑layer of *T. forsythia* are low in healthy individuals, they are significantly elevated in adult and early-onset periodontitis patients. These results do not only indicate that this major surface protein is antigenic in humans, but also suggest that an increased interaction between host adaptive immune mechanisms and this pathogen occurs during periodontal disease progression [37].

Data on the virulence potential of the *T. forsythia* S‑layer were also supported in our laboratory by investigating the immune responses of human macrophages and gingival fibroblasts upon stimulation with wild-type *T. forsythia* and an S-layer-deficient mutant [28]. This mutant induced significantly higher levels of the proinflammatory mediators IL-1b, TNF-a, and IL-8 compared with wild-type cells, especially at the early phase of response. This suggests that the S-layer attenuates the host immune response to this pathogen by evading its recognition by the innate immune system of the host [28]. It will be interesting to see, whether this finding can be confirmed in an animal model.

In a very recent study, first insights into the impact of *Tannerella* cell surface glycosylation on the modulation of the host immunity could be obtained [50]. By comparing the immunological effects evoked with *T. forsythia* wild-type and a Δ*wecC* mutant (see Section 2.2.2. and Section 6.2.), in which the terminal sugar motif consisting of the two subterminal Man*p*NAcA and 4-Me*O*-b-Man*p*NAcCONH_2_ residues and the terminal Pse5Am7Gc residue are missing [29], it became evident that the mutant is less virulent in a periodontitis model [50]. There are indications that the glycan decoration on *Tannerella* cells has a role in suppressing Th17-mediated neutrophil infiltration in the gingival tissue, allowing pathogen persistence in the host and induction of disease [50].

## 4. Cultivation and growth of *T. forsythia*

*T. forsythia* can be cultivated under anaerobic conditions at 37 °C in tryptic soy broth, supplemented with yeast extract (5 g/L), phytone peptone (5 g/L), cysteine (0.2 g/L), horse serum (20 mL/L), hemin (2.5 µg/mL), menadione (2 g/mL), and *N*-acetylmuramic acid (10 µg/mL) [28].

*N*-acetylmuramic acid, which is the monomeric form of the bacterial cell wall component, is an important growth-stimulating factor for *T. forsythia*, both in broth and plate culture [56]. Grown on agar media in presence of *N*-acetylmuramic acid, *T. forsythia* cells appear as regularly-shaped, short, Gram-negative rods, while in the absence of *N*‑acetylmuramic acid growth is retarded and *T. forsythia* cells appear large, filamentous and pleomorphic with tapered (fusiform) ends [9,14]. Since *T. forsythia* lacks a metabolic pathway to synthesize *N*-acetylmuramic acid, the bacterium may possess unique systems to scavenge peptidoglycan degradation products released during cell-wall recycling of oral biofilm bacteria [24]*,* or it derives this compound from sialylated glycoproteins like salivary mucins and fibronectin present in the oral cavity [57].

Because of its unique growth requirements [5] and the fact that it is quite difficult to grow, the precise role of *T. forsythia* in the severe bone and tissue destruction at sites from which it can be isolated remains to be determined.

## 5. Glycosidic Activity of *T. forsythia*

Possibly linked to its asaccharolytic nature, *T. forsythia* possesses genes for at least eight different glycosidases, including sialidases, an a-glucosidase, a b-glucosidase, a fucosidase, an arabinosidase, a glucosaminidase, a galactosidase and a mannosidase; which are able to process terminal glycosidic linkages of the complex oligosaccharides and proteoglycans of the periodontium [26]. This degradation creates a pool of accessible sugars for uptake and nutrition of oral bacteria and affects the functional integrity of the periodontium. Thus, glycan-interaction based processes, such as movement of leukocytes to the site of infection, could potentially be hindered, while protein epitopes for bacterial adhesion are created, further promoting disease progression [24,58]. In this context, an *N*‑acetylneuraminyllactose-sensitive hemagglutinin has been identified in *T. forsythia* [59], which may mediate bacterial binding to host cell-surface sugars exposed by bacterial glycosidases [24].

One class of enzymes that is active on these host molecules are sialidases which represent a family of glycosylhydrolases that cleave a‑ketosidic linkages between sialic acid and the glycosyl residues of host glycoproteins, glycolipids or colominic acid. Recent evidence suggests that for several periodontal pathogens, but particularly for the ‘red complex’ organism *T. forsythia*, sialic acid‑containing host molecules play an important role *in vivo* [60]. Two different sialidases have been found to be expressed in *T. forsythia*, SiaHI and NanH [61]. Previously, Ishikura *et al.* [25] cloned the *siaHI* gene from *T. forsythia* ATCC 43037. The enzyme is found in a variety of cells, including viruses, bacteria, protozoa, fungi, and metazoans. In the case of *T. forsythia*, no definite function has yet been attributed to this enzyme, although experiments point to it being a periplasmic protein that plays no role in extracellular interactions [61]. These same studies also indicated that mutants of the NanH sialidase, the higher expressed sialidase in *T. forsythia*, had hindered attachment and invasion capabilities on epithelial cells. The enzyme was later on seen to play an important role in biofilm growth on surfaces coated with salivary glycoproteins [62]. Furthermore, sialic acid, glycolylsialic acid, and sialyllactose, all of which are common sugar moieties on a range of important host glycoproteins, were seen to stimulate growth of the pathogen only when cultured as a biofilm. The *nanH* gene is located in a large cluster that expands over a 16-kb section of its genome. This cluster contains all the genes required for sialic acid catabolism, which indicates that the cleaved sialic acid can additionally be up-taken and utilized [62].

The sialic acid catabolism cluster shows strong sequence and genome organization similarity to sialic acid loci of related gastrointestinal anaerobes and represents a new route differing from the *Escherichia coli* paradigm pathway for sialic acid use [60]. Taken together, these data indicate that sialic acid is a key growth factor for *T. forsythia* and may be the key to its physiology *in vivo*.

## 6. Biofilm Life-Style of *T. forsythia*

### 6.1. General Remarks

In its native environment of the oral cavity, *T. forsythia* is present in a biofilm, which, in turn, is crucial for the virulence potential of the bacterium. Thus, the knowledge of factors triggering biofilm formation might reveal valuable strategies for interfering with periodontal disease. Despite *T. forsythia* being a late colonizer intercalating with other species from the oral microflora in dental plaque biofilms [63], which is consistent with a polymicrobial disease etiology [24], investigation of monospecies biofilms can also shed light onto factors affecting this specific life style.

### 6.2. Biofilm Life-Style of *T. forsythia* and Glycosylation

Several studies evaluating the presence of *T. forsythia* in subgingival plaque have demonstrated a significantly higher frequency in diseased subjects compared to healthy controls [9]. Moreover, *T. forsythia* was frequently associated with colonization by *P. gingivalis* and was elevated in groups of older patients [64]. Since biofilms formed by periodontal bacteria are considered important in disease progression and pose difficulties in treatment, the investigation of the underlying mechanism of the *T. forsythia* biofilm formation has been initiated [48]. This was carried out by screening random insertion mutants of *T. forsythia* for alterations in biofilm development. The approach led to the identification of a Δ*wecC* mutant [48], with *wecC* encoded in the predicted S‑layer glycosylation gene locus [29]. This mutant showed increased cell surface hydrophobicity, which would promote bacterial attachment and/or aggregation [48] and increased ability to form biofilms as compared to the parent strain.

The truncated S‑layer oligosaccharide isolated from the *T. forsythia* Δ*wecC* mutant represents a partial structure of the above described S‑layer oligosaccharide, in which the acidic branch is missing (see Section 3.2) [29]. Considering a pK value of sialic acids of ~2.6, it is evident that under physiological conditions of the basic saliva environment in the oral cavity, the acid function of Pse5Am7Gc is dissociated and, thus contributes to charge repulsive forces which impair biofilm formation. The negative correlation between *wecC* transcription and biofilm formation [48] supports this assumption. However, since no truncated S‑layer glycans could be detected on *T. forsythia* wild-type cells when grown under biofilm conditions, it is conceivable to assume that the specific glycosylation status of the cell surface is a means to balance the tendency for biofilm-formation at a certain level *in vivo*.

In this context it is interesting to note that, while *wecC* is down-regulated, the *T. forsythia* S‑layer genes *tfsA* and *tfsB* as well as the genes encoding the glycoproteins TF1259 and TF2339 are up-regulated in biofilm formation [65]. This is a further indication of the S‑layer protein O‑glycosylation system to be linked with the biofilm life-style of *T. forsythia*.

## 7. Conclusions

*T. forsythia* is a Gram-negative oral pathogen for which sialic acid is a key growth factor that may be crucial for its physiology *in vivo*. Understanding the biology of oral pathogens and their virulence factors is a prerequisite for the maintenance of both general and oral health. Especially those factors that are associated with the bacterial cell envelope and/or exposed to the environment are prime candidates for mediating virulence through their direct involvement in pathogen-host interactions. Given that *T. forsythia* emerged as a crucial periodontal pathogen [4,9,24,28,60], several approaches were undertaken to characterize its cell surface properties including S‑layer topography and glycosylation [29,35,38,39]. Considering the fact that this bacterium can affect systemic health, *T. forsythia* and especially its mechanisms governing pathogenicity deserve detailed investigation.

Recently, detailed microscopic, biochemical, and molecular analyses from our laboratory revealed that the outer membrane of *T. forsythia* is covered with a so far unique S‑layer. The monolayer has a width of approximately 22 nm and is built up by the co-assembly of the glycosylated S-layer proteins TfsA and TfsB [39]. The *O*-glycosidically linked *T. forsythia* S‑layer oligosaccharide is an overall highly diverse structure containing several rare sugar residues [29] contradicting the so far valid building plan of bacterial S-layer glycans [66,67], and, thus, being reminiscent of archaeal S-layer glycans [68]*.* It is tempting to speculate that the terminal Pse5Am7Gc residue participates in the bacterium-host cross-talk, although the relevance of the modification of the pseudaminic acid remains yet unclear. This notion is supported by the fact that members of this class of sialic acid-like sugars have been found in many Gram-negative bacterial species as constituents of important cell surface glycoconjugates, such as LPS [69], capsules [70], pili [71], and flagella [72,73], all of which are important mediators of pathogenicity, possibly influencing bacterial adhesion, invasion, and immune evasion [74]. It seems plausible that the glycans are recognized by lectin-like receptors that may facilitate adhesion to and invasion of specific host cells [55]. In this context it is interesting to note that a novel sialic acid utilization and uptake system has been described for *T. forsythia* [56]. This opens the possibility of using the sialic acid pathway for biosynthesis of Pse5Am7Gc. On the other hand, it is also possible that the pseudaminic acid might be prone to at least partial degradation by the sialidase system [60].

Crucial for the overall virulence potential of *T. forsythia* is its specific biofilm life-style. Thus, the knowledge of factors triggering biofilm formation might reveal valuable strategies for interfering with periodontal disease. Surprisingly, increased biofilm formation could be correlated with the presence of truncated S-layer glycans on *T. forsythia*, in which the “acidic” branch of the decasaccharide is missing. In the context of biofilm formation, it is interesting to note that besides the *T. forsythia* S‑layer glycoproteins, two other glycoproteins that have been identified recently [29], are up-regulated in biofilm formation [65]. This is a further indication that the S-layer protein *O*‑glycosylation system is linked with the biofilm life-style of *T. forsythia*. The finding that several abundant proteins in *T. forsythia* are modified with the S-layer glycan (as shown in Figure 2) is supported by the recent identification of a rich outer membrane glycoproteome in *T. forsythia* [45]. Our data corroborate and extend this study by Veith *et al.* [45], in which all but one protein (TF0091) have already been identified as glycoproteins.

Thus, the periodontal pathogen *T. forsythia* possesses a general protein *O*-glycosylation pathway that modifies proteins of yet undefined function at multiple sites with a complex oligosaccharide within the D(S/T)(A/I/L/M/T/V) amino acid motif. The underlying glycosylation machinery as well as the glycosylation ‛sequon’ seems to be conserved within *Bacteroidales* species [46]. The role of protein *O*‑glycosylation in underpinning the pathogenic strategy of *T. forsythia* and in its interaction with other bacteria from the oral microflora will be the subject of future studies.
